# European Journal of Medical Research reviewer acknowledgment 2014

**DOI:** 10.1186/s40001-015-0111-y

**Published:** 2015-02-25

**Authors:** Dieter Häussinger, Johannes Waltenberger

**Affiliations:** Department of Internal Medicine, University of Düsseldorf, D-40225 Düsseldorf, Germany; Department of Cardiovascular Medicine, University of Münster, 48149 Münster, Germany

## Abstract

The Editors of *European Journal of Medical Research* would like to thank all of our reviewers who have contributed to the journal in Volume 19 (2014)

**Abdel Razek Ahmed**

Egypt

**Addeo Raffaele**

Italy

**Agarwal Shiv Kumar**

United States

**Ahmad Umraan**

United States

**Akdemir Ovunc**

Turkey

**Aniuliene Rosita**

Lithuania

**Antic Darko**

Serbia

**Arnold Paul**

United States of America

**Arnold Tim**

Germany

**Artz Gregory J**

United States of America

**Asensi Victor**

Spain

**Assaf Naglaa**

Egypt

**Aytac Erman**

Turkey

**Banke Ingo**

Germany

**Barad David**

United States of America

**Beirer Marc**

Germany

**Bek Yuksel**

Turkey

**Berner Arne**

Germany

**Bevc Sebastjan**

Slovenia

**Bhushan Premanshu**

India

**Bignami Elena**

Italy

**Bin Song**

China

**Binder Thomas**

Germany

**Bold Richard**

United States of America

**Borgia Guglielmo**

Italy

**Bosch-Barrera Joaquim**

Spain

**Bostjancic Emanuela**

Slovenia

**Brandalize Ana Paula**

Brazil

**Brehm Antonio**

Portugal

**Breithardt Ole-A**

Germany

**Browning Robert**

United States of America

**Butera Gianfranco**

Italy

**Cai Lu**

United States of America

**Campbell Brewster**

Germany

**Caputo Massimo**

United Kingdom

**Cardoso Rhanderson**

United States of America

**Carvalho Fernanda**

Brazil

**Casini Arianna**

Italy

**Casso Dominguez Abel**

United States of America

**Castro Pablo**

Chile

**Cavaliere Franco**

Chile

**Cavlak Ugur**

Turkey

**Cetisli Korkmaz Nilufer**

Turkey

**Chen Yong**

China

**Chen Jiankui**

China

**Chihara Norio**

United States of America

**Christensen Robin**

Denmark

**Colagreco Joseph**

United States of America

**Cuesta Fernandez Ana**

United States of America

**Delhey Patrick**

Germany

**Deng Zhong-Liang**

China

**Diez Diego**

Japan

**Doudican Nicole**

United States of America

**Dragoumis Dimitrios**

United Kingdom

**Drenth Joost**

United States of America

**Economopoulos Konstantinos**

United States of America

**Ehrmann Stephan**

France

**Epiphanio Sabrina**

Brazil

**Fabiani Emiliano**

Italy

**Fenoglio Luigi**

Italy

**Frink Michael**

Germany

**Furuta Mayuko**

Japan

**Gaofeng Li**

China

**Gentile Ivan**

Italy

**Gill Jennifer Li**

Germany

**Goldberg Robert**

United States of America

**Gonzalez Andrew**

United States of America

**Greiner Jack**

United States of America

**Guo Lin**

China

**Gutiérrez-Venegas Gloria**

Mexico

**Han Ping**

China

**Hanschen Marc**

Germany

**Hayati Abd Rahman**

Malaysia

**Heggermont Ward**

Belgium

**Henrich Dirk**

Germany

**Hernández Luis**

Spain

**Hevezi Peter**

United States of America

**Hida Toyoaki**

Japan

**Hoff Norman-Philipp**

Germany

**Hu Xiaoyun**

China

**Hu Jian**

Hong Kong

**Huang Chi-Ying**

Taiwan

**Huang DaWei**

Armenia

**Huang Rongchong**

China

**Huang Wei**

China

**Hüneke Bernd**

Germany

**Imbeaud Sandrine**

France

**Irving-Rodgers Helen**

Australia

**Izbicki Jakob**

Germany

**Janowskiq John**

United Kingdom

**Jennings Jessica**

United States of America

**Jiang Tao**

China

**Jin Lingtao**

United States of America

**Juhnn Yong-Sung**

South Korea

**Jurisic Vladimir**

Serbia

**Kaiser Gernot**

Germany

**Kammers Kai**

United States of America

**Kawachi Hiroshi**

Japan

**Keck Tobias**

Germany

**Kemona Halina**

Poland

**Keymel Stefanie**

Germany

**Kim Tae Hyun**

South Korea

**Kimble Roy**

Australia

**Kitagawa Masanobu**

Japan

**Kleinbongard Petra**

Germany

**Klostermann Cyrus**

Germany

**Kogut Michael**

United States of America

**Komm Nadja**

Germany

**Kosugi Shin-ichi**

Japan

**Kröpil Patric**

Germany

**Landoni Giovanni**

Italy

**Li Gang**

China

**Li Xiaojian**

United States of America

**Li Yuhua**

China

**Li Jiangtao**

China

**Lichte Philipp**

Germany

**Lin Haijiang**

United States of America

**Lipinski Marek**

Poland

**Liu Delong**

United States of America

**Liu Gongjun**

China

**Liverneaux Philippe**

France

**Lorand-Metze Irene**

Brazil

**Lorenz Kerstin**

United States of America

**Luk Ivi**

Poland

**Luo Xianghang**

China

**Lustig Livia**

Argentina

**Luton Dominique**

France

**Ma Lin**

China

**Ma Feng**

China

**Magalhaes Marco**

United States of America

**Maisch Bernhard**

Germany

**Major Eugene**

United States of America

**Makhoul Imad**

Israel

**Manyazewal Tsegahun**

Ethiopia

**Maofeng Wang**

China

**Marinucci Lorella**

Italy

**Maselli Diego**

United States of America

**Masroor Imrana**

Pakistan

**Matuschek Christiane**

Germany

**Mavroudis Dimitris**

Greece

**Mei Qibing**

China

**Meyer Christian**

Germany

**Meyer zu Hörste Gerd**

Germany

**Mihai Radu**

United Kingdom

**Miller Ashley M.**

American Samoa

**Miller Robert**

United Kingdom

**Mor Yoram**

Israel

**Mossad Sherif**

United States of America

**Motoyama Satoru**

Japan

**Muellenbach Ralf**

Germany

**Muler Kersen**

Germany

**Müller Christian W.**

Germany

**Nakabayashi Kazuhiko**

Japan

**Namas Rajaie**

United States of America

**Narod Steven**

Canada

**Nashan Björn**

Germany

**Neder Luciano**

Brazil

**Neuhaus Thomas**

Germany

**Niu Yinbo**

China

**Nixdorff Uwe**

Germany

**Noutsias Michel**

Germany

**Nowak-Markwitz Ewa**

Poland

**Noyez Luc**

Netherlands

**Oette Mark**

Germany

**Packthongsuk Kreeson**

Thailand

**Palomeque Julieta**

Argentina

**Parratt John**

Australia

**Pascual Angela M**

Spain

**Patel Achint**

United States of America

**Pavenski Katerina**

Canada

**Pawlik Andrzej**

Poland

**Peiper Matthias**

Germany

**Perlini Stefano**

Italy

**Petretta Mario**

Italy

**Pin Richard**

United States of America

**Pineau Pascal**

France

**Plewka Danuta**

Poland

**Pontieri Francesco**

Italy

**Qin Lun-Xiu**

China

**Qiu Haibo**

China

**Ramírez-Velázquez Carlos**

Mexico

**Rey Johannes**

Germany

**Ridder Dirk De**

Canada

**Rodriguez Juan Carlos**

Spain

**Rodríguez-Ruiz David**

Spain

**Rohrbach Marianne**

Switzerland

**Rosado Balmayor Elizabeth**

Germany

**Rotunno Melissa**

United States of America

**Sakka Samir**

Germany

**Samra Jaswinder**

Australia

**Schenke-Layland Katja**

Germany

**Schick Martin**

Germany

**Seeliger Claudine**

Germany

**Segat Ludovica**

Italy

**Seow Kok-Min**

Taiwan

**Sethi Jigme**

United States of America

**Seubert Bastian**

Germany

**Severi Carola**

Italy

**Shimada Hideaki**

Japan

**Shulman Lee**

United States of America

**Sikorska Katarzyna**

Poland

**Song Yong**

China

**Sonstegard Tad S**

Australia

**Spears Norah**

United Kingdom

**Strachan Paul**

United States of America

**Svensson Per-Arne**

Sweden

**Sze Sing-Hoi**

United States of America

**Takeda Akihiro**

Japan

**Tang Zi-Hui**

China

**Tang Albert**

China

**Tappia Paramjit**

Canada

**Tartaglia Edoardo**

Italy

**Thomson Rebecca**

Australia

**Tillou Xavier**

France

**Tiwari Nidhish**

United States of America

**Tomaluma RC**

Japan

**Tong Mingjie**

United States of America

**Tsoutsou Pelagia**

Greece

**Tuomisto Anne**

Finland

**Van Griensven Martijn**

Germany

**Vancem Jeffery**

United Kingdom

**Vaneckova Ivana**

Czech Republic

**Vassiou Katerina**

Greece

**Verhoeven Adrie**

Netherlands

**Wallerman Ola**

Sweden

**Walocha Jerzy**

Poland

**Wan Song**

Hong Kong

**Wang Pu**

China

**Wang Wen**

China

**Wang Luhua**

China

**Wang Wei-Ming**

China

**Weig Thomas**

Germany

**Werdan Karl**

United Kingdom

**Wu Zhifeng**

China

**Xu Zhen**

United States of America

**Xu Renshi**

China

**Yannarelli Gustavo**

Argentina

**Yao Yong-ming**

China

**Yarali Hakan**

Turkey

**Yin Leimiao**

China

**Yip Cheng-Har**

Malaysia

**Yoshida Takafumi**

Japan

**You Yongping**

China

**You Zongbing**

United States of America

**Yu Zhangsheng**

United States of America

**Yuchen Fan**

China

**Zeng Zihua**

United States of America

**Zeng Mian**

China

**Zhang Lin**

China

**Zhang Yanyun**

China

**Zhang Sen**

China

**Zhou Linuo**

China

**Zhu Chunfu**

China

**Ziaei Maryam**

Australia

